# Supplementary material for: The Win–Win Effects of an Invasive Plant Biochar on a Soil–Crop System: Controlling a Bacterial Soilborne Disease and Stabilizing the Soil Microbial Community Network
Source: Microorganisms. 2024 Feb 22;12(3):447. doi: 10.3390/microorganisms12030447 (PMC10972153; doi:10.3390/microorganisms12030447)
Supplement: Supplementary file 1 [file microorganisms-12-00447-s001.zip › microorganisms-2865990-supplementary.pdf]

The Win–Win Effects of an Invasive Plant Biochar on a Soil–Crop System: Controlling a Bacterial Soilborne Disease and Stabilizing the Soil Microbial Community Network

Sheng Wang [1,2,3], Lei Wang [1,2,3], Sicong Li [1,2,3], Tiantian Zhang [4] and Kunzheng Cai [1,2,3,*]

[1] College of Natural Resources and Environment, South China Agricultural University, Guangzhou 510642, China; wangsheng@stu.scau.edu.cn (S.W.); kishi218@163.com (L.W.); 2021lsc@stu.scau.edu.cn (S.L.)
[2] Key Laboratory of Tropical Agricultural Environment in South China, Ministry of Agriculture and Rural Affairs, South China Agricultural University, Guangzhou 510642, China
[3] Guangdong Provincial Key Laboratory of Eco-Circular Agriculture, South China Agricultural University, Guangzhou 510642, China
[4] College of Horticulture, South China Agricultural University, Guangzhou 510642, China; 17863608557@163.com
* Correspondence: kzcai@scau.edu.cn; Tel.: +86-20-38297175

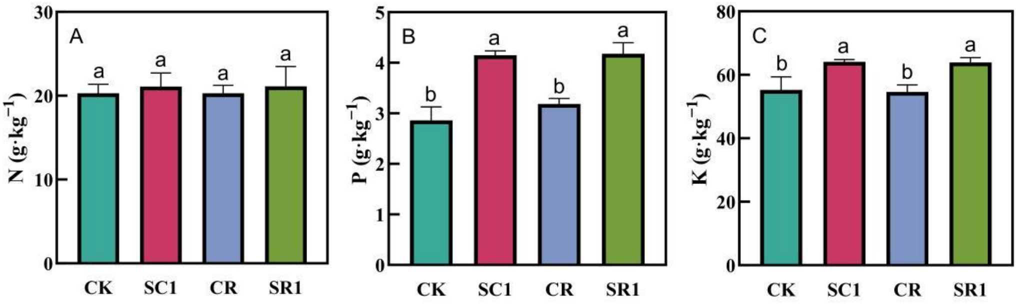

Figure S1. Effects of invasive plant biochar treatments on N(**A**), P(**B**) and K(**C**) uptake in tomato plants. CK, SC1 represent 0%, 1% biochar application without pathogen inoculation, respectively; CR, SR1 represent 0%, 1% biochar application with pathogen inoculation, respectively. All values are presented as the mean ± standard error (n = 3). Different letters in treatments indicate significant differences (P < 0.05).

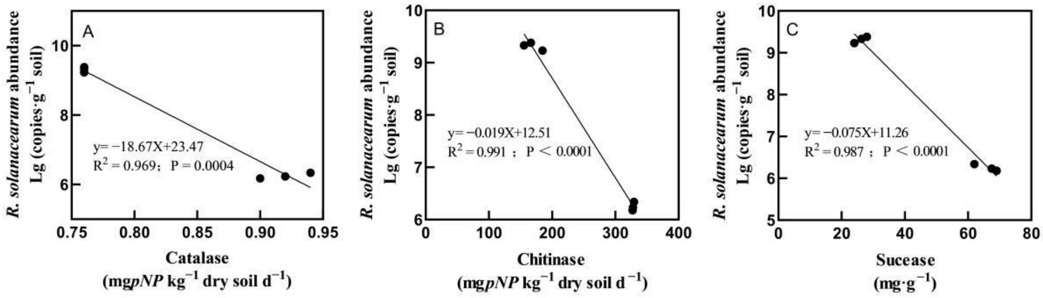

Figure S2. Correlation analysis of soil enzyme activities and the abundance of *R. solanacearum* in soil. Catalase (**A**), Chitinase **(B)**, Sucrase (**C**).

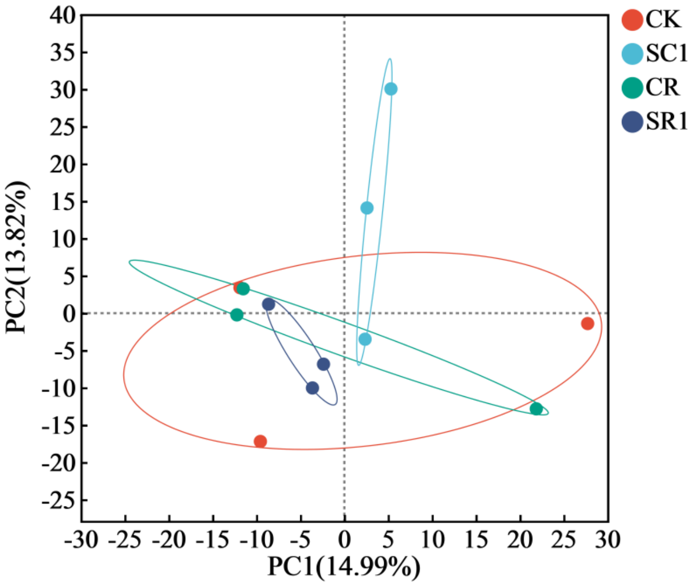

Figure S3. Effects of invasive plant biochar treatments on soil bacterial community structure. CK, SC1 represent 0%, 1% biochar application without pathogen inoculation, respectively; CR, SR1 represent 0%, 1% biochar application with pathogen inoculation, respectively.

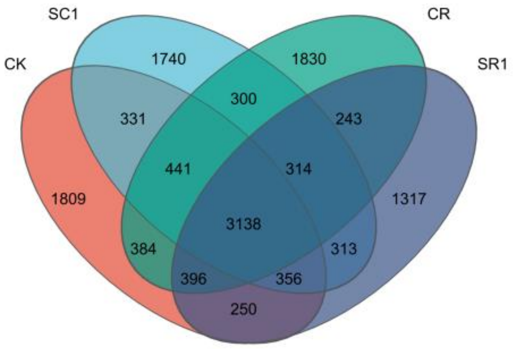

Figure S4. Effects of invasive plant biochar treatments on soil microbial diversity. CK, SC1 represent 0%, 1% biochar application without pathogen inoculation, respectively; CR, SR1 represent 0%, 1% biochar application with pathogen inoculation, respectively.

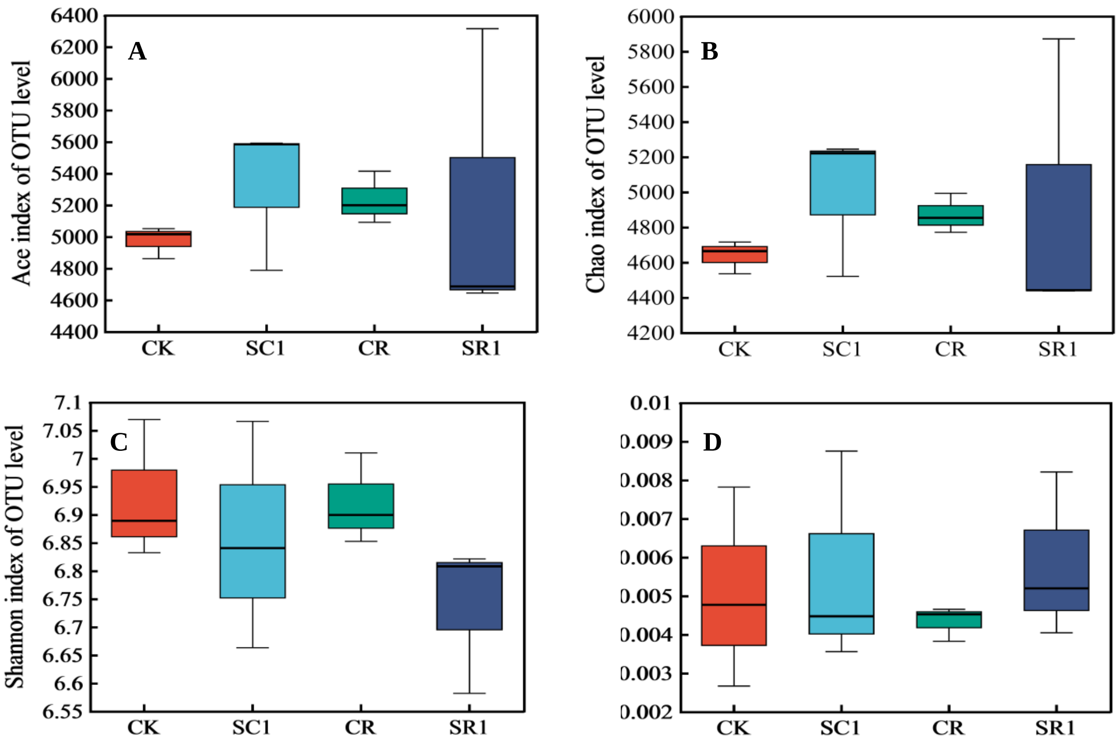

Figure S5. Effects of invasive plant biochar treatments on soil microbial diversity. Ace index (**A**), Chao index (**B**), Shannon index (**C**), Simpson index (**D**). CK, SC1 represent 0%, 1% biochar application without pathogen inoculation, respectively; CR, SR1 represent 0%, 1% biochar application with pathogen inoculation, respectively. All values are presented as the mean ± standard error (*n* = 3).
